# Supplementary material for: Assembly and Comparison of Ca. Neoehrlichia mikurensis Genomes
Source: Microorganisms. 2022 May 31;10(6):1134. doi: 10.3390/microorganisms10061134 (PMC9227406; doi:10.3390/microorganisms10061134)
Supplement: Supplementary file 1 [file microorganisms-10-01134-s001.zip › Supplementary Figure S1,2.pdf]

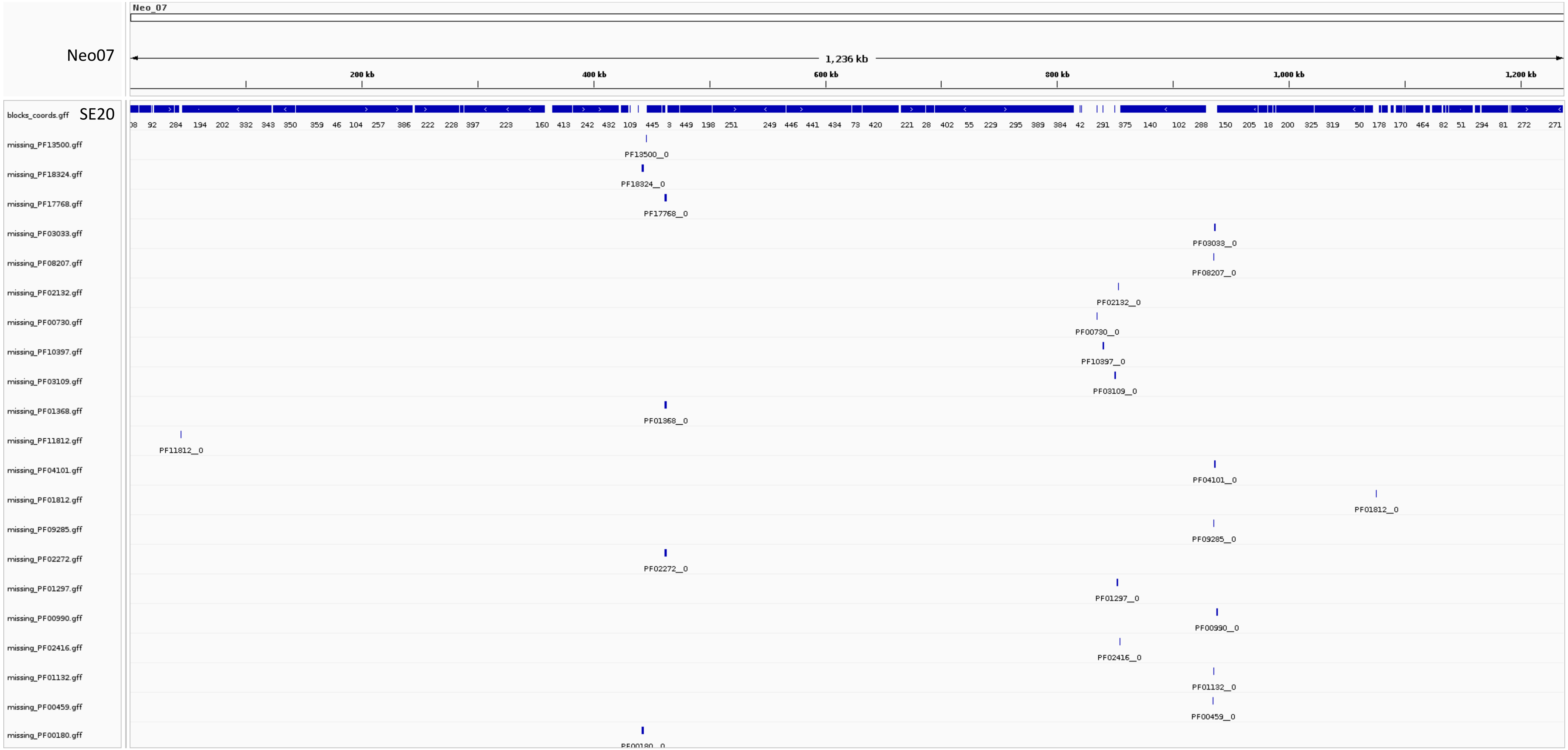

Supplementary Figure S1: Visualized using IGV shows the gaps found in the Swedish genome SE20 and the genes that fill those gaps in Neo07.

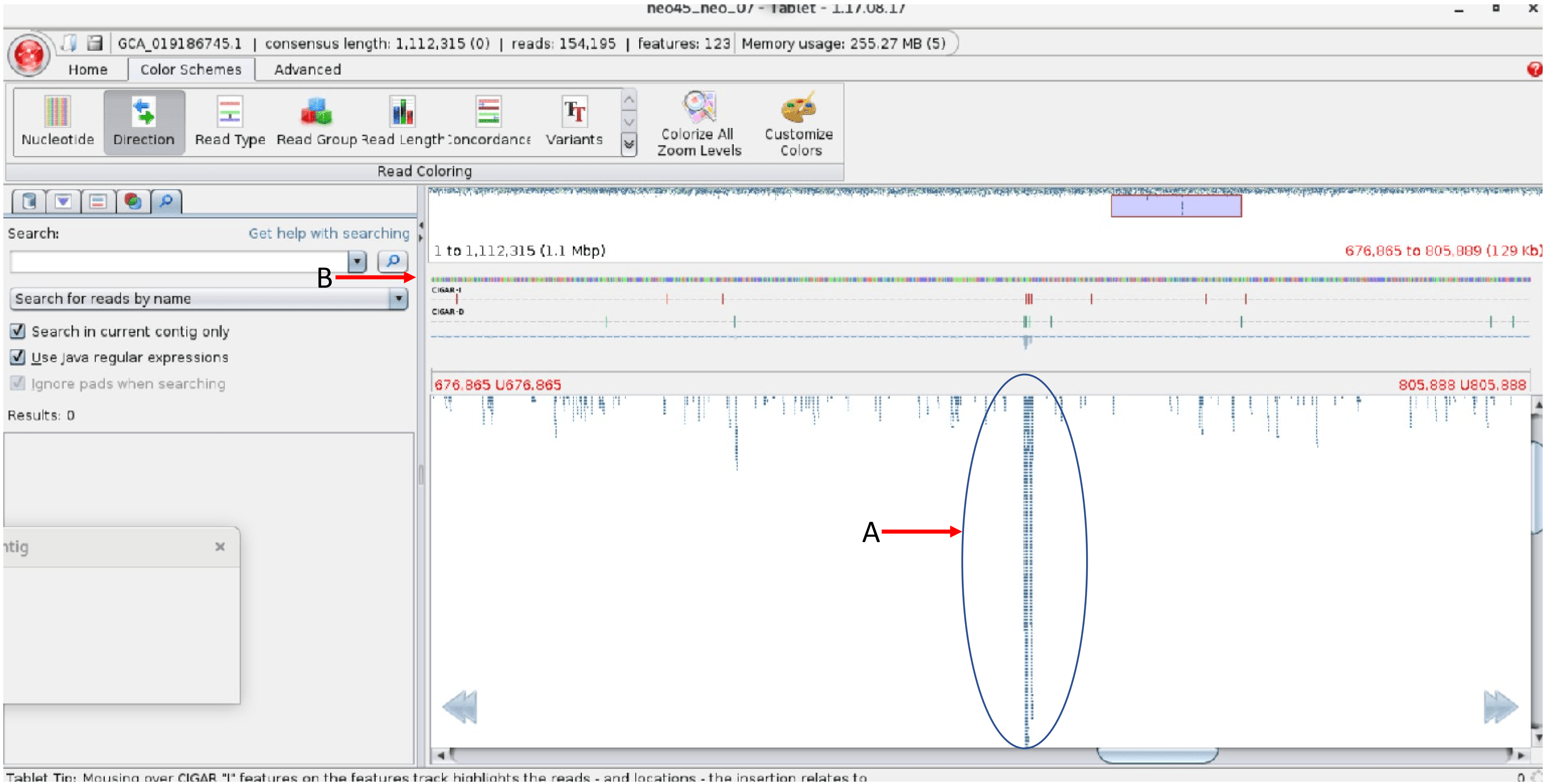

Supplementary Figure S2: Neo_07 Illumina reads were aligned to the published Swedish genome SE20 using Minimap2. As visualized with Tablet, there is an accumulation of reads (A) encoding the PF01617 outer membrane domain that stack on top of the SE20 assembly (B) in the region around 743000-755000bp.
